# Antiseizure properties of fenamate NSAIDs determined in mature human stem-cell derived neuroglial circuits

**DOI:** 10.3389/fphar.2024.1385523

**Published:** 2024-05-16

**Authors:** Hamed Salmanzadeh, Robert F. Halliwell

**Affiliations:** Thomas J. Long School of Pharmacy, University of the Pacific, Stockton, CA, United States

**Keywords:** multi electrode array, epilepsy, mefenamic acid, cyclooxygenase inhibitors, flufenamic acid, GABAA receptors

## Abstract

Repeated and uncontrolled seizures in epilepsy result in brain cell loss and neural inflammation. Current anticonvulsants primarily target ion channels and receptors implicated in seizure activity. Identification of neurotherapeutics that can inhibit epileptiform activity and reduce inflammation in the brain may offer significant benefits in the long-term management of epilepsy. Fenamates are unique because they are both non-steroidal anti-inflammatory drugs (NSAIDs) and highly subunit selective modulators of GABA_A_ receptors. In the current study we have investigated the hypothesis that fenamates have antiseizure properties using mature human stem cell-derived neuro-glia cell cultures, maintained in long-term culture, and previously shown to be sensitive to first, second and third generation antiepileptics. Mefenamic acid, flufenamic acid, meclofenamic acid, niflumic acid, and tolfenamic acid (each tested at 10–100 μM) attenuated 4-aminopyridine (4-AP, 100 μM) evoked epileptiform activity in a dose-dependent fashion. These actions were as effective diazepam (3–30 μM) and up to 200 times more potent than phenobarbital (300–1,000 μM). The low (micromolar) concentrations of fenamates that inhibited 4-AP evoked epileptiform activity correspond to those reported to potentiate GABA_A_ receptor function. In contrast, the fenamates had no effect on neural spike amplitudes, indicating that their antiseizure actions did not result from inhibition of sodium-channels. The antiseizure actions of fenamates were also not replicated by either of the two non-fenamate NSAIDs, ibuprofen (10–100 μM) or indomethacin (10–100 μM), indicating that inhibition of cyclooxygenases is not the mechanism through which fenamates have anticonvulsant properties. This study therefore shows for the first time, using functionally mature human stem cell-derived neuroglial circuits, that fenamate NSAIDs have powerful antiseizure actions independent of, and in addition to their well-established anti-inflammatory properties, suggesting these drugs may provide a novel insight and new approach to the treatment of epilepsy in the future.

## Introduction

Fenamate NSAIDs including mefenamic acid, flufenamic acid, tolfenamic acid and niflumic acid, are broad spectrum cyclooxygenase (I and II) inhibitors with antipyretic, anti-inflammatory and analgesic properties (Hill and Zawia, 2021). This class of NSAID also uniquely modulates GABA_A_ receptors in a highly subunit-dependent manner (Halliwell et al., 1999; Mansikkamäki et al., 2019). Work from this lab, for example, first reported that mefenamic acid potentiates GABA_A_-receptor mediated currents recorded from recombinant human α1β2γ2 receptors expressed in *Xenopus* oocytes and HEK-293 cells but inhibits GABA currents recorded from α1β1γ2 GABA_A_ receptors in these expressions systems (Halliwell et al., 1999). Similarly, niflumic acid potentiates recombinant rat α1β3γ2 GABA currents recorded from *Xenopus* oocytes but inhibits α1β1γ2 GABA mediated responses (Mansikkamäki et al., 2019). Point mutation experiments and molecular modeling show that potentiation of GABA_A_ receptors by fenamates occurs via an allosteric site in the transmembrane 2 domain of the β2 and β3 subunits shared with the general anaesthetic, etomidate and the antiseizure agent, loreclezole (Halliwell et al., 1999; Mansikkamäki et al., 2019).

Native GABA_A_ receptor mediated currents recorded from cultured rat hippocampal neurons (Coyne et al., 2007) and cerebellar Purkinje neurons (Rossokhin et al., 2019) are also potentiated by fenamates. Significantly, the low micromolar concentrations of fenamates that potentiate GABA currents do not have effects at glutamate, NMDA or glycine receptor mediated responses (Coyne et al., 2007) but higher (10^4^ to 10^3^ molar) concentrations of fenamates can have pleiotropic actions at diverse ion channel and receptor sites in neural and non-neuronal cells (Guinamard et al., 2013).

Fenamates pass the blood brain barrier (Bannwarth et al., 1989; Gee et al., 2010; Subaiea et al., 2011), suggesting that their actions at the major inhibitory receptor in the mammalian brain may evoke central nervous system effects. Moreover, their efficacy and ability to potentiate GABA_A_ receptors parallels the sedative-hypnotic and anticonvulsant agents including the benzodiazepines and barbiturates. Consistent with this notion, in cases of human overdose, mefenamic acid is associated with altered mental status and lethargy (Doğan et al., 2019), coma (Gossinger et al., 1982; Hendrickse, 1988) or convulsions (Smolinske et al., 1990). Notably, in animal studies, mefenamic acid was reported to inhibit convulsions evoked by the muscarinic receptor agonist, pilocarpine (Ikonomidou-Turski et al., 1988) and by the GABA_A_ receptor antagonist, pentylenetetrazol (Wallenstein, 1991).

Diverse preclinical and clinical studies show that cyclooxygenases are induced during seizures and that inhibition of these inflammatory enzymes using NSAIDs can reduce seizure recurrence and disease severity and increase the efficacy of conventional anticonvulsants (Radu et al., 2017; Rawat et al., 2019). Fenamate NSAIDs, with GABA_A_ modulating properties, might thus hold significant value in the development of primary and/or adjunctive treatment of patients with epilepsy. In the present study, therefore, we have addressed the hypothesis that fenamate NSAIDs have antiseizure properties by utilizing human stem cell-derived neuron-glial cells maintained in long-term culture. We have recently reported that this 2D *in vitro* model forms complex excitatory (*glutamatergic*) and inhibitory (*GABAergic*) neural circuits over many weeks, paralleling *in vivo* development, with synaptogenesis and electrophysiological activity facilitated by the secondary development of astrocytes in the cultures; significantly, we have also shown that these human neuronal cell networks generate epileptiform activity in response to diverse convulsant agents and to be highly sensitive to the anticonvulsant actions of first, second and third generation antiepileptic drugs (Salmanzadeh et al., 2023).

## Methods

### Stem cell culture and differentiation

Pluripotent human (TERA2. cl.SP12) stem cells were maintained and differentiated as previously described (e.g., Salmanzadeh et al., 2023). Briefly, non-differentiated stem cells were cultured in media consisting of Dulbecco’s Modified Eagles Medium (DMEM, Sigma–Aldrich), 10% Fetal Bovine Serum (FBS, Gibco), 2 mM L-glutamine (Gibco) and penicillin–streptomycin (100 U/mL, 100 μg/mL; Invitrogen), and maintained in an incubator at 37°C, 5% CO_2_, 100% relative humidity in 75 mL tissue culture flasks until 80% confluent. Cells were then re-seeded on pre-coated glass coverslips for immunocytochemistry experiments, or MEA plates for electrophysiology and neuropharmacology studies. To differentiate stem cells towards neurons and glia, retinoic acid (10 μM; Sigma-Aldrich) was added to the culture media, which was refreshed every 2–3 days.

### Immunocytochemistry

Briefly and as previously described, cells for immunocytochemistry were cultured on 18 mm diameter sterile glass coverslips, precoated with poly-D-lysine (50 μg/mL; Sigma-Aldrich) at a density of 50,000 cells/cm^2^. For labeling, cells were fixed with 4% formaldehyde, permeabilized in 0.3% Triton X-100 and placed in blocking solution comprised of 5% normal donkey serum for 1 h at ambient room temperature; thereafter they were incubated in primary conjugated antibody (Santa Cruz Biotechnology) for βIII tubulin (1:100) or glial fibrillary acidic protein (GFAP, 1:100) at 4°C overnight. Cells were subsequently counter-stained with the nuclear dye, DAPI (100 ng/mL; Sigma-Aldrich) and mounted on chamber slides with anti-fade solution (Prolong1 Gold Antifade Reagent; Invitrogen). The cells were later viewed and imaged using an inverted EVOS FL auto microscope (Thermo Fisher Scientific) and the βIII tubulin^+^ neurons or GFAP^+^ glial cells calculated as a percentage of the total number of DAPI^+^ cells in 5 separate fields (0.23 mm^2^ per field) for each slide.

### Multi-electrode array recording

Electrophysiological recordings were conducted from human stem cell-derived neuroglia cultures using the Maestro system and AxIS software (Axion Biosystems) as previously described (Salmanzadeh et al., 2023). In the current study, three 6-well MEA plates were utilized. Each well contained 64 PEDOT electrodes, arranged in an 8 × 8 grid and covering a recording area of 2.1 mm × 2.1 mm; an MEA plate therefore contained a total of 384 electrodes.

Three different stocks of human stem cells, cultured and differentiated at different times over the course of 1 year, contributed to the electrophysiological data set reported here. Stem cells were plated at 200,000 cells per well in the MEA plates (Cytoview MEA, Axion Biosystems), pre-coated with polyethyleneimine (Sigma-Aldrich) and maintained at 37°C, 5% CO_2_, 100% relative humidity. Neural cultures were maintained by exchanging 50% of the culture media with fresh, pre-warmed media every 3 days.

The MEA plates were placed into the Maestro Edge platform and equilibrated for 10 min prior to a baseline recording period of at least 10–15 min. Electrical signals were sampled at 12.5 kHz, with low and high pass filters set between 200Hz and 3KHz; the spike threshold was computed with an adaptive threshold of 6 times the estimated rms noise on each channel. All recordings were performed at a constant temperature of 37°C and 5% CO_2_. The RAW data files were subsequently re-recorded with AxIS software to convert the electrophysiological signals into Microsoft Excel files that included spike timing and profile information. To be included in our data analysis, only electrodes with activity of 5 or more spikes per minute over the recording time were included. AxIS Navigator software was then used to determine 1) the number of electrodes/well with activity of ≥5 spikes/min, 2) the *Weighted Mean Firing Rate* from all active electrodes, 3) the number of *Single Electrode Bursts*, which are clusters of at least 5 spikes from an electrode, with a maximum inter-spike interval (ISI) of 100 ms; 4) the number of Network Bursts which are defined as a minimum of 50 spikes, with an ISI of ≤100 ms and included at least 35% of the electrode array participating in a 20 ms time epoch; and (v) the Synchrony Index which is derived from a cross-correlogram of spikes occurring at times relative to each other across all unique pairs of electrodes. Synchrony values lie between 0 and 1, with those closer to 1 indicating higher synchrony and coordinated neural activity (see Halliday et al., 2006).

### Drug solutions

Stock solutions of drugs were freshly prepared and filtered using 0.22 μM solvent resistant filters (Millipore) as follows: mefenamic acid, flufenamic acid, niflumic acid, tolfenamic acid, indomethacin and ibuprofen (all from Sigma-Aldrich) were dissolved in DMSO; diazepam was dissolved in ethanol, Aminopyridine (4-AP); phenobarbital and meclofenamic acid sodium salt were dissolved in water to obtain stock solutions of 10 mM.

### Drug testing procedures

4-AP is a well-established convulsant agent both *in vivo* and *in vitro* (Ventura-Mejía et al., 2023) and we have previously shown that it is highly effective in provoking epileptiform activity in our stem cell-derived neuroglial cultures (Salmanzadeh et al., 2023). Control spontaneous firing was therefore recorded for a minimum of 10 min, 4-AP (100 μM) then added to the cells and activity was recorded for a further 60 min. This was followed by addition of either flufenamic acid (10–100 μM), mefenamic acid (10–100 μM), meclofenamic acid (10–100 μM), niflumic acid (10–100 μM) or tolfenamic acid (10–100 μM). To compare the efficacy and potency of the fenamate NSAIDs on epileptiform activity in the human neuron 2D cultures, we also tested the actions of diazepam (3–30 μM) and phenobarbital (100–1000 μM). We then determined the impact of the non-fenamate NSAIDs, indomethacin (10–100 μM) and ibuprofen (10–100 μM). All drugs were washed out once an asymptotic change had been established and the cells replenished with fresh culture media. Cells were then placed back into the CO_2_ incubator. Our pharmacological experiments were conducted on cells differentiated between 40 and 50 weeks *in vitro*.

### Statistical analysis

Calculation of means, standard error of means (SEM), 95% Confidence Intervals (95%CI) and graphical presentation of the data were conducted using GraphPad Prism software (Version 10). Inferential statistical analysis was limited to Two-Way Analysis of Variance (ANOVA) and Dunnett’s *post hoc* tests of neural spike amplitude, single electrode (cell) bursts, network bursts and synchrony index (a measure of coordinated firing across the neural network) in the absence and presence of experimental drugs. Statistical significance was accepted at *p* ≤ 0.05.

## Results

### Neuroglial cultures

**FIGURE 1:**
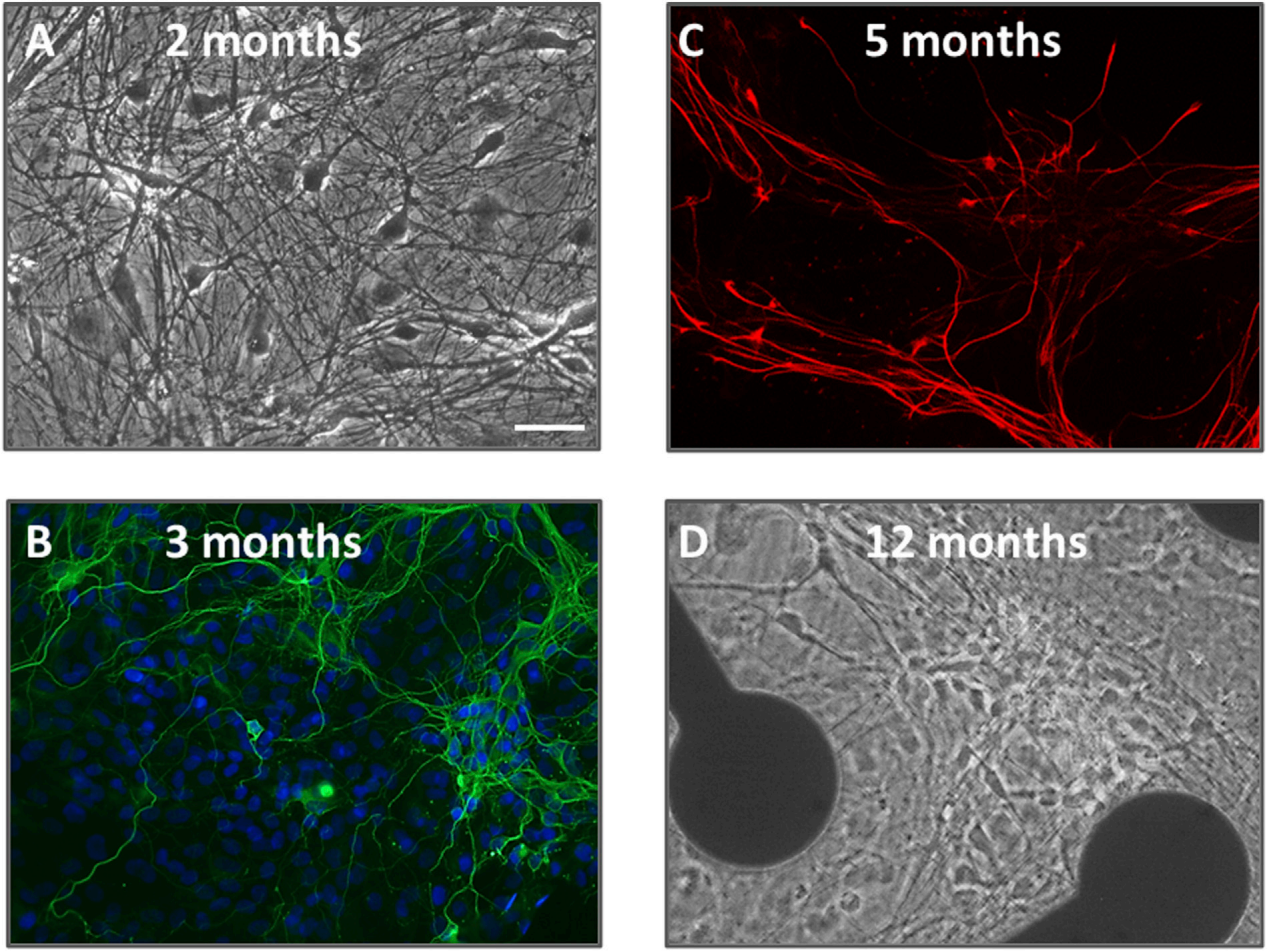
A snapshot of human neurons and glial cells over 12 months in culture utilized in this study. **(A,D)** are phase-contrast photomicrographs of cells at 2 months and 12 months neural differentiation. Pyramidal and multipolar neurons are visible with extensive axodendritic processes. In the image at 12 months, cells are shown on an MEA plate with 2 of the 64 electrodes visible. **(B)** is an immunofluorescence image of neurons at 3 months *in vitro*, fixed and stained with the nuclear dye DAPI (blue) plus β-III tubulin (green); **(C)** is an immunofluorescence image of glial cells at 5 months fixed and labeled with glial-fibrillary acidic protein (GFAP, red). The scale bar = 25 μm and applies to all panels.

Human [TERA2.cl.SP12] stem cell derived neurons and glial cells were successfully differentiated and maintained for up to 88 weeks in cell culture. As previously detailed, neurons and glia differentiated and matured over the course of 4–6 months with increased axodendritic networks and synapse formation continuing through 12 months (Salmanzadeh et al., 2023). Figure 1 shows a *snapshot* of neurons and glia in phase contrast (Figures 1A, D) and immunolabeled (Figures 1B, C) over the course of year 1. Neuroglial cultures maintained between 40 and 50 weeks *in vitro* were used in this study, and were comprised of a stable number of neurons and glial cells (indicated by β-III tubulin and GFAP labeling) and displayed high and stable firing patterns (see Salmanzadeh et al., 2023).

### Fenamate NSAIDs and antiseizure properties

As we have previously described, MEA recordings show that individual neurons are spontaneously active within 2–4 weeks of the start of neural differentiation, fire single and later bursts of action potentials and groups of neurons generate network bursts (through excitatory and inhibitory synaptic circuits) and synchronized firing patterns (Salmanzadeh et al., 2023). Neural activity in our neuroglial cultures increased up to 10 months and was well maintained through 20 months of culture time.

**FIGURE 2:**
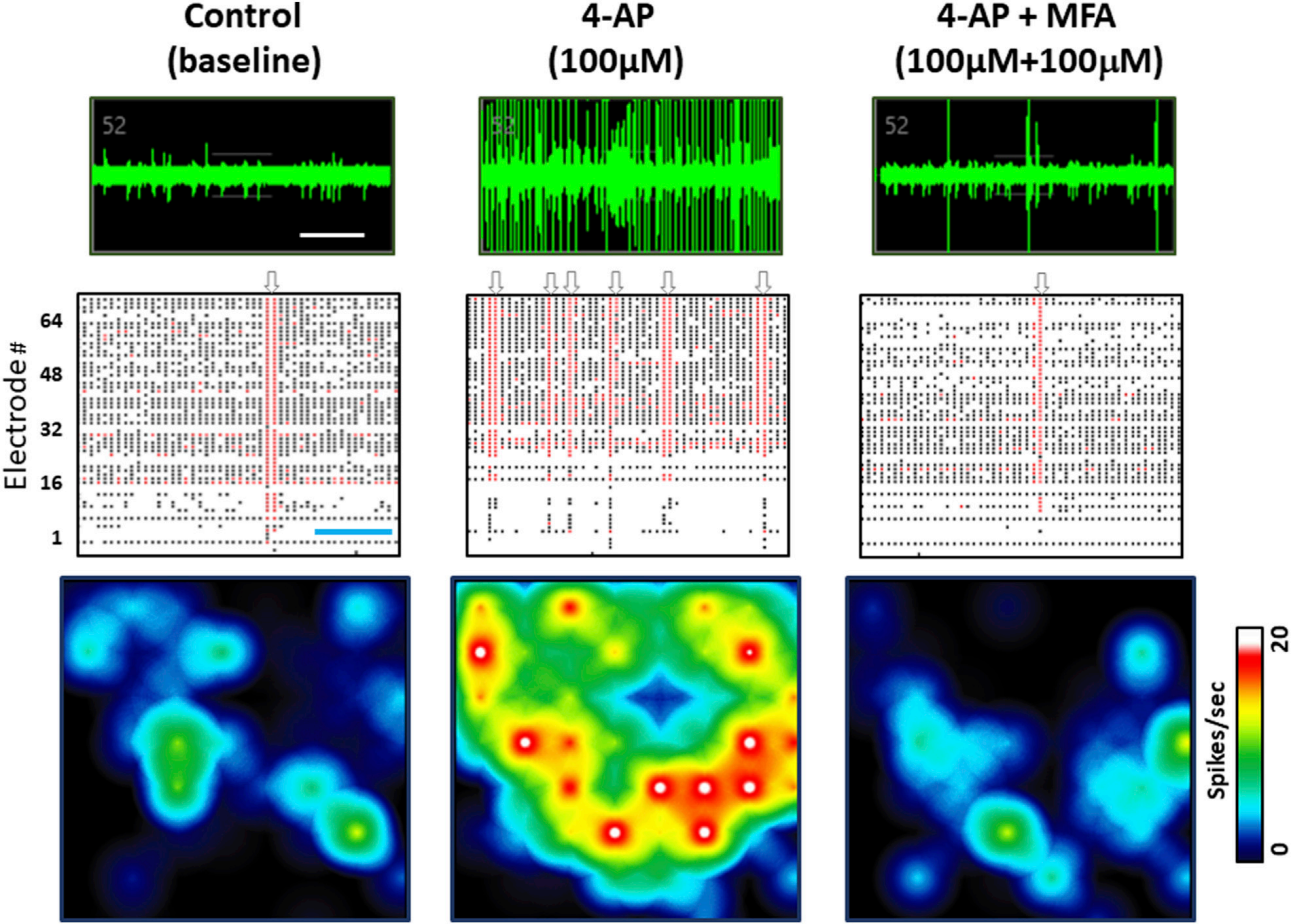
Mefenamic acid Inhibits 4-AP evoked epileptiform activity. Top row shows MEA voltage recordings from a single human stem cell-derived neuron firing spontaneous action potentials, shown as spikes on the trace in (left) control media; (center) the presence of 4-AP (100 µM); (right) 4-AP (100 µM) +mefenamic acid (MFA 100 μM). Individual spikes and several single cell bursts can be seen, especially in the 4-AP voltage trace. Middle rows show Raster plots of spike events, represented by the black lines, single cell bursts indicated by the red lines, and network bursts indicated by the grey arrows above each panel. Each Raster plot represents approximately 100 s of recording time (300 s in total) and shows that 1 network burst was observed in the control period, six network bursts in the presence of 4-AP, and only 1 burst in the presence of 4-AP+mefenamic acid. Bottom row shows color-coded heatmaps from a six well MEA plate with blue indicating lower (*circa* 1Hz) spike frequency and red/white higher (20Hz) individual cell firing rates. Note that single cell and network bursts greatly increase in the presence of 4-AP (100 µM) but are attenuated in the presence of MFA (100 μM). The time calibration line in the voltage trace is 10 s and, in the Raster plot, it is 25 s.

**FIGURE 3:**
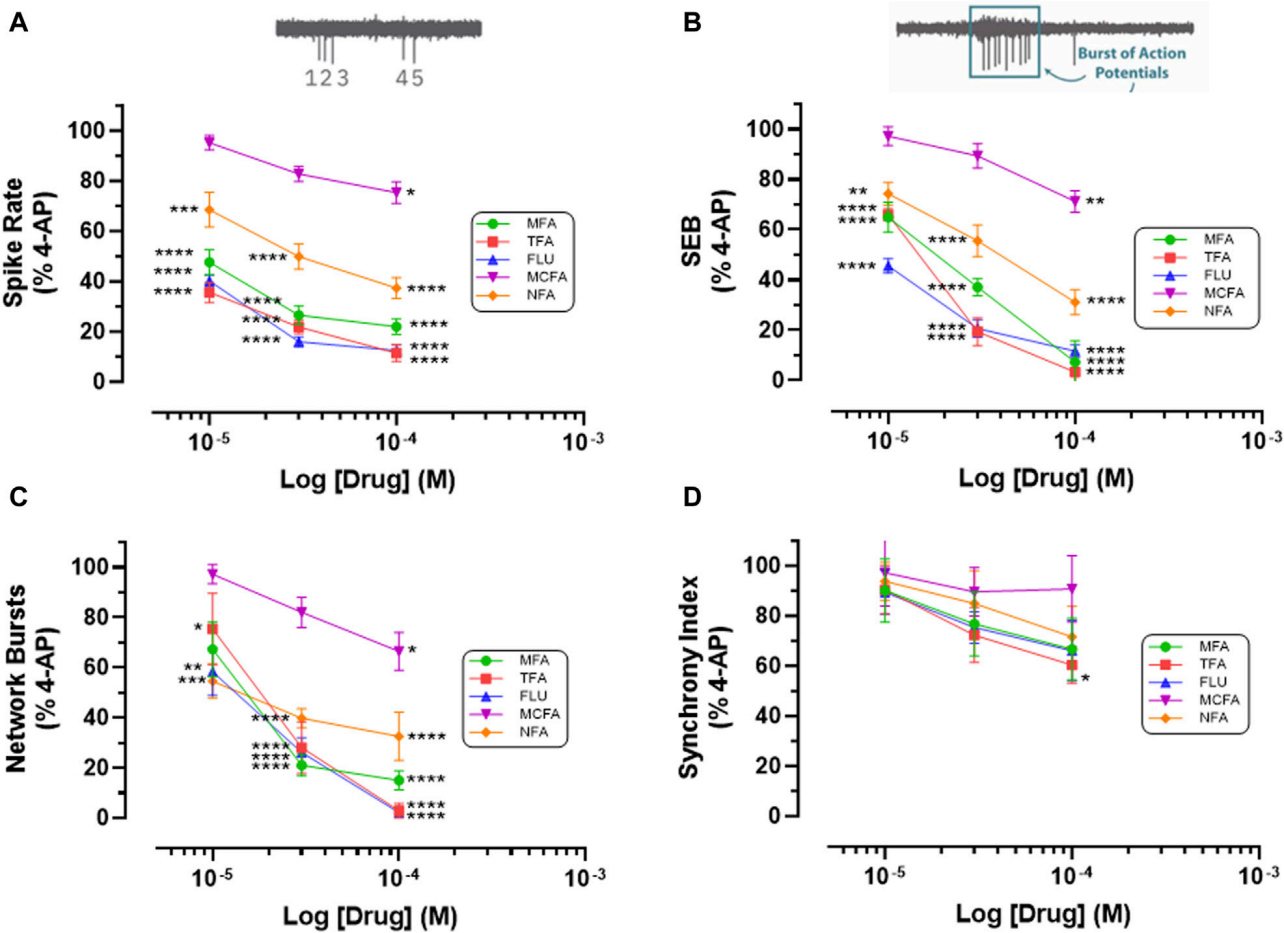
Fenamates Inhibit 4-AP evoked epileptiform activity. The graphs show that mefenamic acid (MFA), tolfenamic acid (TFA), flufenamic acid (FFA), meclofenamic acid (MCFA) and niflumic acid (NFA) significantly (*) attenuate 4-AP-evoked increases in **(A)** spike rate, **(B)** single cell bursts (SEB = Single Electrode Burst on y-axis), and **(C)** network bursts in a concentration dependent manner; tolfenamic acid (100 μM) also significantly reduced **(D)** synchronized firing. The log drug concentration is shown on the x-axis and the response, as % of activity in the presence of 4-AP (100 μM), is shown on the y-axis. The effect of all drugs reversed upon washout. The insert above **(A)** is a simple schematic of 5 single spikes and above **(B)** a schematic of a burst of 9 action potentials. *p ≤ 0.05; **p ≤ 0.002; ***p ≤ 0.001; ****p ≤ 0.0001.

Our previous study showed that anticonvulsants, including diazepam and phenobarbital reduced spontaneous spike rate in these neuronal and glial cell cultures (Salmanzadeh et al., 2023). Therefore, to address the hypothesis that fenamates may have antiseizure properties, we first tested the effects of mefenamic acid (1–100 μM) on baseline neural spike activity. Consistent with the GABA_A_ receptor potentiators (diazepam and phenobarbital) mefenamic acid reduced the spontaneous spike rate in a concentration-dependent fashion to 99% ± 1%, 86% ± 4% and 67% ± 5% (*n* = 4) of control at 1 µM, 10 µM and 100 µM, respectively. Secondly, we determined the effects of mefenamic acid (10–100 μM) on 4-aminopyridine (4-AP, 100 μM) induced epileptiform neuronal activity which, as we have previously reported, evokes a highly significant increase in spike rate, single cell bursts, network bursting and synchronized firing (see Supplementary Figure S1). As can be seen in Figure 2, mefenamic acid attenuated epileptiform activity by reducing spike rate, single cell bursting, and network bursts when compared with the solvent control. Moreover, inhibition of epileptiform activity by mefenamic acid was concentration dependent and highly significant, as shown in Figure 3.

Thirdly, we addressed the question of whether the antiseizure effects of mefenamic acid was a unique property of this drug or representative of the fenamate class by testing the effects of flufenamic acid, meclofenamic acid, niflumic acid and tolfenamic acid (each at 10–100 μM) against 4-AP-evoked epileptiform-activity in our stem-cell-derived neuron-glia circuits. Notably, and like mefenamic acid, all fenamates significantly reduced seizure-like neural spiking, single and network bursting in a concentration-dependent manner when compared with their solvent controls; tolfenamic acid (100 μM) also reduced synchronized firing patterns (see Figure 3). As can be seen, tolfenamic acid, flufenamic acid and mefenamic acid were the most potent, reducing 4-AP-evoked increases in spike rate by over 50% at 10 μM.

### Neural spikes, fenamates, GABA_A_ modulators and non-fenamate NSAIDs

To address the hypothesis that the fenamates reduce seizure-like events by inhibiting sodium channels, we then analyzed their impact on neural spike amplitudes and compared them with the GABA_A_ potentiators, diazepam and phenobarbital in our seizure model. As can be seen in Table 1, neither the addition of 4-AP (100 μM) nor the combination of any of the five fenamates (all tested at 100 μM) with 4-AP (100 μM) changed the neural spike amplitudes; similarly, neither of the control GABA_A_ potentiators, diazepam (10 μM) or phenobarbital (1 mM) inhibited the neural spike amplitudes.

**TABLE 1:** Summarizes the impact of the fenamates and the GABA receptor potentiators on the neural spike amplitudes.

Drug	Spike Amp (µV) Control (mean ± SEM)	Spike Amp (µV) in 4-AP (mean ± SEM)	Spike Amp (µV) in 4-AP+Drug (mean ± SEM)
Mefenamic Acid	42 ± 2	46 ± 4	45 ± 3
Tolfenamic Acid	44 ± 4	46 ± 7	44 ± 5
Flufenamic Acid	42 ± 3	43 ± 5	43 ± 3
Meclofenamic	39 + 4	41 ± 5	43 ± 5
Niflumic Acid	38 ± 3	42 ± 4	40 ± 4
Diazepam	38 ± 3	42 ± 5	37 ± 5
Phenobarbital	41 ± 4	43 ± 5	40 ± 5

Above is shown the amplitude of the action potentials (*spikes*) in the absence of drugs (control), in the presence of 4-AP (100 μM) and in the presence of 4-AP (100 μM) plus each of the fenamates (tested at 100 μM), diazepam (10 μM) or phenobarbital (1 mM). Two-way analysis of variance and *post hoc* comparisons confirmed that none of these conditions significantly affected the neural spike amplitudes.

**FIGURE 4:**
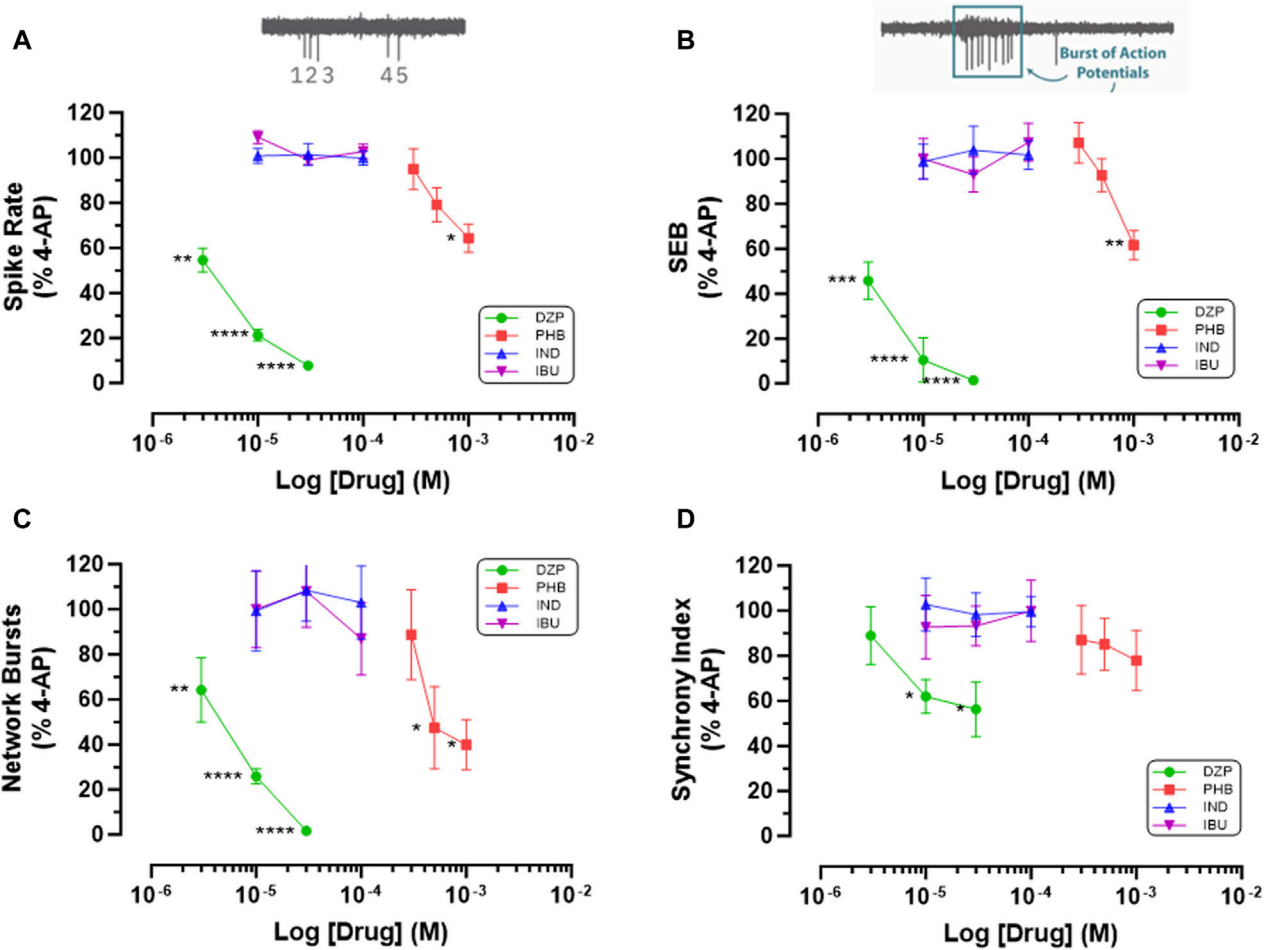
Non-fenamate NSAIDs do not Inhibit 4-AP evoked epileptiform activity. The graphs show that neither indomethacin (10–100 μM) nor ibuprofen attenuate **(A)** 4-AP-evoked spike rate, **(B)** single cell bursts, **(C)** network bursts and **(D)** synchronized firing. In contrast in the same stem cell-derived neuroglial cultures, diazepam (3–30 μM) and phenobarbital (300–1,000 μM) significantly (*) inhibited epileptiform activity in a concentration-dependent manner. The log drug concentration is shown on the x-axis and the response as % of activity in the presence of 4-AP (100 μM) is shown on the y-axis. The effect of all drugs reversed upon washout. The insert above **(A)** is a simple schematic of 5 single spikes and above **(B)** a burst of 9 action potentials. *p ≤ 0.05; **p ≤ 0.002; ***p ≤ 0.001; ****p ≤ 0.0001.

We next investigated the hypothesis that cyclooxygenase inhibition underlies the antiseizure properties of fenamates by testing the two non-fenamate NSAIDs, indomethacin and ibuprofen (which do not modulate GABA_A_ receptors) and compared them with the GABA_A_ potentiators, diazepam and phenobarbital (that do not have anti-inflammatory properties) on epileptiform behaviour in our neuro-glial cell networks. As can be seen in Figure 4, neither ibuprofen (10–100 μM) nor indomethacin (10–100 μM) had any effect on 4-AP (100 μM)-evoked epileptiform spiking, single or network burst firing or the level of synchronized activity. In contrast, in these same cells, diazepam (3–30 μM) and phenobarbital (300–1000 μM) significantly inhibited epileptiform activity in a highly concentration-dependent manner compared with their solvent controls (Figure 4). These latter observations are consistent with an extensive literature, both *in vitro* and *in vivo*, that potentiation of GABA_A_-mediated synaptic transmission has powerful anti-epileptiform actions. In addition, our data now show that the antiseizure effects of the fenamates NSAIDs are not the result of the inhibition of cyclooxygenases, since neither of the non-fenamate NSAIDs had any effect against 4-AP-evoked epileptiform activity.

## Discussion

To our knowledge, fenamates are unique because they are both anti-inflammatory and highly subunit-selective modulators of GABA_A_ receptors at low micromolar concentrations (Halliwell et al., 1999; Mansikkamäki et al., 2019; Rossokhin et al., 2019). These NSAIDs also cross the blood brain barrier indicating that they have central nervous system actions. Consistent with this hypothesis, mefenamic acid attenuates the cognitive impairments of beta amyloid in rodent models of Alzheimer’s disease (Joo et al., 2006; Daniels et al., 2016; Hill and Zawia, 2021), improves cognitive function in patients undergoing androgen deprivation treatment for prostate cancer (Melnikov et al., 2021), reduces depressive-like symptoms in mice subjected to chronic mild stress (Feng et al., 2020) and is neuroprotective in rats against transient middle cerebral artery occlusion (Khansari and Halliwell, 2019). Likewise, flufenamic acid improves neurologic outcome in mice subjected to cardiac arrest followed by cardiopulmonary resuscitation (Chen et al., 2022).

Our present study now demonstrates that fenamates also have powerful antiseizure properties in mature human cultured stem cell derived neuroglial circuits. Notably, four of the fenamates tested were as efficacious as the benzodiazepine, diazepam, and 200 times more potent than the barbiturate, phenobarbital - two globally used anticonvulsants that are also widely available in emergency departments for the rapid termination of *status epilepticus* (Wylie et al., 2024). Consistent with our *in vitro* findings, Ikonomidou-Turski et al. (1988) reported that mefenamic acid “*prevented seizures and protected rats from seizure-related brain damage by pilocarpine*” and Wallenstein (1991) demonstrated that mefenamic acid protected against pentylenetetrazol-induced clonic convulsions in freely moving rats. Since chronic and uncontrolled seizures are associated with brain injury from excitotoxicity and neuroinflammation (Rawat et al., 2019), these data suggest that fenamates may have an important adjunctive role in the management of epilepsy and it is neurological sequalae.

The mechanisms underlying the diverse neurological actions, including the antiseizure effects of the fenamates are not fully understood, but may involve anti-inflammatory and ion channel modulation (e.g., Chen et al., 1998; Cimolai, 2013; Feng et al., 2020; Hill and Zawia, 2021). Preclinical and clinical studies show that some NSAIDs, including aspirin, can reduce the severity and/or frequency of seizures, but only mefenamic acid and flufenamic acid have been shown to inhibit seizure activity *in vivo* (Ikonomidou-Turski et al., 1988; Wallenstein, 1991; Radu et al., 2017; Rawat et al., 2019). Our data now show that fenamates have powerful antiseizure actions in human stem-cell derived neuron circuits and the non-fenamate NSAIDs, indomethacin or ibuprofen do not. Since all of the NSAIDs investigated in this study are inhibitors of cyclooxygenase I and II, our data indicate that the rapid anticonvulsant-like properties of fenamates is not the result of COX enzyme inhibition and a reduction in prostaglandin biosynthesis in neuroglial cells.

Antiseizure actions can result from inhibition of voltage-gated sodium channels (Sills and Rogawski, 2020; Hakami, 2021) and might contribute to the antiepileptic actions of fenamates. In keeping with this idea, mefenamic acid, flufenamic acid and tolfenamic acid inhibit voltage-gated sodium currents (Yau et al., 2010; Sun et al., 2019; Sun et al., 2020) but only at concentrations 10–300 times higher than those required for inhibition of 4-AP evoked epileptiform activity observed in the current study. Moreover, mefenamic acid had little or no effect on the spike amplitudes recorded from our stem cell derived neurons, indicating that sodium ion channel block did not contribute to the antiseizure actions of fenamates. Indeed, the low (μM) concentrations of fenamates that inhibited or completely blocked epileptiform activity were consistent with those that allosterically potentiate GABA_A_-receptor mediated inhibition in central neurons and human recombinant (α1β2γ2) GABA_A_ receptors (Halliwell et al., 1999; Coyne et al., 2007; Dvorzhak, 2008; Mansikkamäki et al., 2019; Rossokhin et al., 2019). In keeping with this hypothesis, the two control GABA_A_ potentiators, diazepam and phenobarbital, inhibited 4-AP evoked epileptiform activity in a manner paralleling that of the fenamates.

Inhibition of excitatory neurotransmission *via* glutamate receptor antagonism is also a potential mechanism through which fenamates could have antiseizure properties. Consistent with this hypothesis, Fernández et al. (2010) reported that flufenamic acid suppressed 4-AP evoked epileptiform activity in rat hippocampal slices by inhibition of glutamate receptors at concentrations of 50–200 μM, and an IC_50_ of 61 μM. Similarly, Lerma and del Rio (1992) showed that niflumic acid (and flufenamic acid) inhibited NMDA currents recorded from rat spinal cord neurons with an IC_50_ of 353 μM, whereas Coyne and coworkers (2007) reported that mefenamic acid had no effect at 100 μM on glutamate or NMDA evoked currents recorded from cultured rat hippocampal neurons. Together these data illustrate that fenamates are 10–100 times less potent antagonists of glutamate receptors than potentiators of GABA_A_ receptors and that glutamate inhibition played little or no part in their antiseizure actions determined in our human stem cell derived neuronal circuits. More critically, the high micromolar concentrations of fenamates that inhibit sodium channels (discussed above) or glutamate receptors, are non-specific with actions on multiple ion channels, transporters and receptors (e.g., Cimolai, 2013; Guinamard et al., 2013) and confound interpretation of the data in these reports.

Finally, a number of alternative mechanisms of action might contribute to the antiseizure properties of fenamates, including interactions at chloride and potassium ion channels (e.g., Peretz et al., 2005; Guinamard et al., 2013) and will require further investigation. However, the very low (micromolar) concentrations that are highly effective in attenuating epileptiform activity correspond closely with those that selectively potentiate GABA_A_ receptors. Notably, potentiation of GABA_A_ receptors by fenamates is highly beta2/3-subunit dependent and parallels the actions of other clinically important modulators including the anticonvulsant loreclezole and the general anaesthetic etomidate (Halliwell et al., 1999; Mansikkamäki et al., 2019). Interestingly, recent studies have now shown that transmembrane accessory proteins, such as Shisa7, which selectively associates with alpha1, alpha 2 and gamma 2 subunits of GABA_A_ receptors, can modulate the actions of diazepam, suggesting additional targets for drug action and therapeutic drug development (Han et al., 2021).

In conclusion, this study adds to the growing evidence that fenamates are a unique class of NSAID *and* subunit selective modulators of GABA_A_ receptors with anti-inflammatory, antipyretic, analgesic, neuroprotective and antiepileptic properties (e.g., Cimolai, 2013; Hill and Zawia, 2021). This well-established class of therapeutic drugs may therefore provide a novel insight and new approach to the treatment and management of epilepsy, stroke and neurodegenerative disorders in the future. Our study also supports the value and power of exploiting mature human stem cell-derived neuron-glial cells, in long-term culture, for the investigation of drug actions on the central nervous system.

## Data Availability

The raw data supporting the conclusion of this article will be made available by the authors, without undue reservation.

## Supplementary material

SUPPLEMENTARY FIGURE S1 4-Aminopyridine (4-AP, 100 μM) induces epileptiform-like activity in mature human stem cell derived neurons: a violin plot showing that 4-AP significantly increased the spike firing rate (SR), the number of individual cell bursts (SEB), the number of network bursts (NB) and the synchrony index (SI). The plot illustrates the distribution of the observations, with individual data points shown within the violin. The center line is the mean (*n* = 7–9) and the top and bottom dotted lines are the 3rd and 1st inter-quartiles, respectively. The data were analyzed using *one sample t-tests*. All 4 electrophysiological parameters were significantly different to control at *p* ≤ 0.001.
