# Supplementary figures and images for: Antiseizure properties of fenamate NSAIDs determined in mature human stem-cell derived neuroglial circuits
Source: Front Pharmacol. 2024 May 16;15:1385523. doi: 10.3389/fphar.2024.1385523 (PMC11141243; doi:10.3389/fphar.2024.1385523)

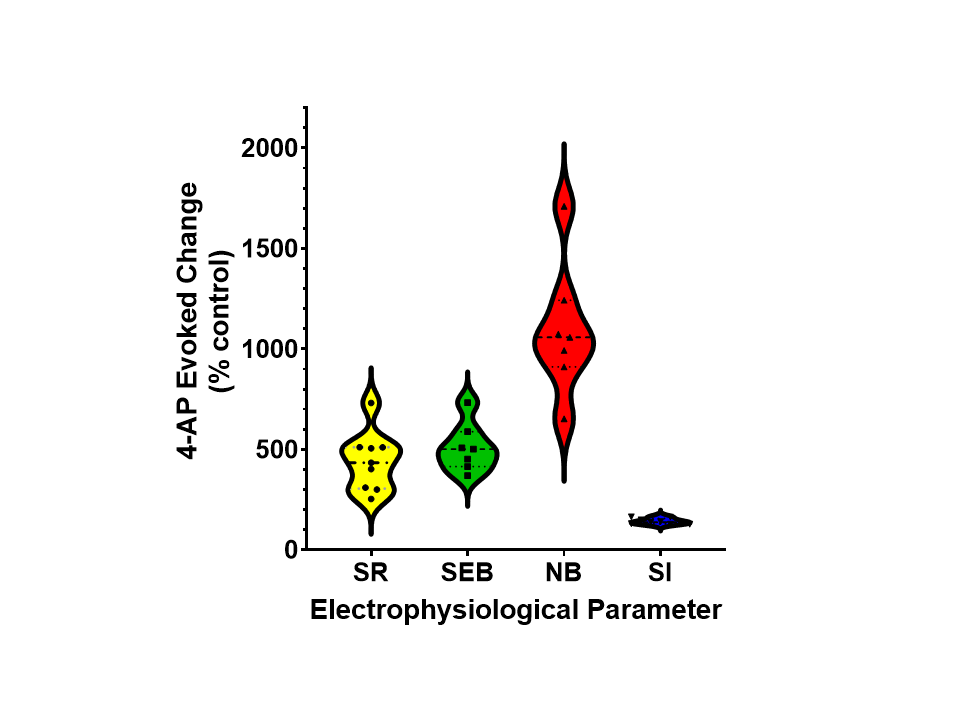

Supplement: Supplementary file 1 [file Image1.TIF]
